# Supplementary material for: Additive Digital Casting: From Lab to Industry
Source: Materials (Basel). 2022 May 11;15(10):3468. doi: 10.3390/ma15103468 (PMC9145986; doi:10.3390/ma15103468)
Supplement: Supplementary file 1 [file materials-15-03468-s001.zip › materials-1670224-supplementary.pdf]

Materials

Supplementary Material 1

Additive Digital Casting: From lab to industry

A preliminary study was carried at ETH to estimate, and possibly improve, the robustness of the mix design used for Digital Fabrication upscaling the concrete production from the laboratory to the industrial scale. The mixing conditions at ETH and the industry facilities are listed in the Table S1.

Table S1: Comparison of the mixing conditions between ETH and industry facilities.

	**ETH laboratory**	**Industry facility**
**Mixer type**	Rotating pan mixer	Counter current mixer
**Mixer configuration**	The mixing star contains four mixing blades and spins in one single rotation speed. A wall scraper is also installed. The mixing pan turns according to the mixing star rotation speed and viscosity of the material.	The mixing pan contains two off side scrapers, which rotate according to the mixing star. Attached to the mixing star two planetary engines, containing two rotating mixing arms, spin with the mixing star.
**Mixing power**	--	2 x 22 kW
**Volume**	40 L	1500 L (for preliminary tests, 500 L)
**Aggregates conditions**	Dry at RT (20 °C)	Humid, cold (stored outside in winter)
**Admixture dosing**	Water and admixture together	Water with powder materials, then admixture

For this purpose, the concrete composition and materials as well as the W/C ratio were kept the same. In contrast, four mixes were produced tuning admixture and accelerator dosages to evaluate the robustness starting from different rheological properties in terms of flow loss and setting time, respectively. The superplasticizer used is BASF Glenium ACE 30 (Switzerland) or SIKA Viscocrete Easy and the accelerator BASF X-SEED 100. The summary of these preliminary comparison mixes are listed in Table S2.

Table S2: Comparison of the mixing conditions between ETH and industry facilities.

	**Slow Flow Loss**	**Fast Flow loss**
**Slow setting**	Mix 3<br>(Glenium ACE 30)	Mix 4<br>(Viscocrete Easy)
**Fast setting**	Mix 1<br>(Glenium ACE 30)	Mix 2<br>(Viscocrete Easy)

Figure S1 shows the evolution of the rheological properties, expressed as spread flow diameters, for the 4 different mixes prepared at ETH (Figure S1 left) and at the industry facility (Figure S1 right). The results of the rheological properties evolution of the mix 3 are also shown in the main text.

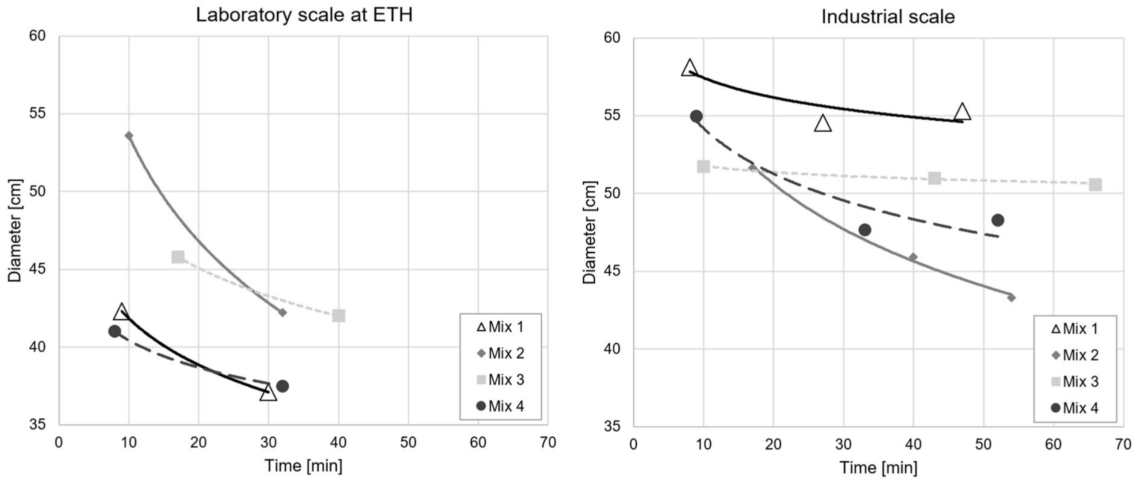

Figure S1: Rheological properties evolution for four mixes prepared (left) at laboratory scale at ETH and (right) at industrial scale.

The mixes produced at larger scale in Figure S1 right highlighted better the main differences given by the change of the admixtures combinations. In fact, as expected, Mix 1 and 3 show a more prolonged workability with respect to the other two mixes, even starting from different initial fluidity values. At laboratory scale, no clear correlation can be identified among the different mixes by changing the admixture combination in terms of fluidity and flow loss (Figure S1 left). In addition, the fluidity is lost much faster in the small scale tests within 30 minutes for all the mixes.

Materials

Supplementary Material 2

Table S3 shows the chemical composition obtained by X-ray fluorescence. Table S4 shows the mineralogical composition of the used cement, as determined by Rietveld analysis of the X-ray diffraction (Bruker AXS D8 ADVANCE with metal target of Cobalt anode) patterns and expressed in values normalized to 100% of crystalline phases.

Table S3 Chemical composition of the used cement Jura Cement I 52.5R and CAC (Ciment Fondue Kerneos). LOI = loss on ignition. IR = insoluble residue. SO_3 and IR contents were determined by gravimetric analysis. Values reported for CAC were included in the product data sheet.

Minerals	Cement Jura I 52.5R % (w/w)	CAC (Ciment Fondu) % (w/w)
CaO	63.5	35.5 - 39.0
SiO_2	20.0	3.5 – 5.5
Al_2O_3	4.9	37.5 – 41.0
MgO	1.8	< 1.5
MnO	0.1	n.a.
SO_3	3.2	< 0.5
Fe_2O_3	3.3	13.0 – 17.5
Na_2O	0.2	n.a.
K_2O	0.7	n.a.
TiO_2	0.3	< 4.0
P_2O_5	0.1	n.a.
LOI	2.0	n.a.
IR	0.5	n.a.

Table S4. Mineralogical composition of the used cement Jura Cement I 52.5R and CAC (Ciment Fondue Kerneos).

Minerals	Cement Jura I 52.5<br>% (w/w)	CAC (Ciment Fondu)<br>% (w/w)
C_3S	64.7	--
C_2S	14.5	8.9
C_3A	5.9	--
C_4AF	8.5	21.4
Gehlenite (C2AS)	--	2.8
Calcium Mono-Aluminate (CA)	--	63.1
Mayenite ($C_{12}A_7$)	--	3.8
Periclase	0.3	--
Gypsum	3.2	--
Hemihydrate	2.3	--
Anhydrite	0.6	--
Calcite	0.1	--

Table S5 reports the mean value and the corresponding standard deviation of the characteristic particle diameter D_{v50} (volume based) of the used materials, measured (in isopropanol suspensions) by laser diffraction with a Malvern Mastersizer 5 instrument (Malvern Instruments Ltd., Malvern, UK). The same table also shows its specific surface area (SSA_{BET}) determined by nitrogen adsorption with a Tristar II instrument by Micromeritics (Micromeritics GmbH, Aachen, Germany). Anhydrous samples were degassed in an external degassing station (VacPrep 061, also by Micromeritics) at 40 °C under nitrogen flow for 16 h.

Table S5 Characteristic particle diameters (volume based) and SSA_{BET} of used materials.

**Material**	**D_{v50} (um)**	**SSA_{BET} (m^2/g)**
Jura Cement I 52.5	9.8	1.0
CAC	13.7	0.76
Fly Ash Type F	1.04	--
$CaCO_3$ Nekafill 15 -KFN	0.82	--
